# Evaluation of the Use of Compressed Sensing in Data Harvesting for Vehicular Sensor Networks

**DOI:** 10.3390/s20051434

**Published:** 2020-03-06

**Authors:** Juan Antonio Martinez, Pedro Miguel Ruiz, Antonio F. Skarmeta

**Affiliations:** 1 Research & Innovation Department, Odin Solutions, 30820 Murcia, Spain; 2 Department of Information and Communications Engineering, University of Murcia, 30100 Murcia, Spain; pedrom@um.es (P.M.R.); skarmeta@um.es (A.F.S.)

**Keywords:** compressed sensing, vehicular sensor networks, gathering data

## Abstract

We propose a new harvesting approach for Vehicular Sensor Networks based on compressed sensing (CS) technology called Compressed Sensing-based Vehicular Data Harvesting (CS-VDH). This compression technology allows for the reduction of the information volume that nodes must send back to the fusion center and also an accurate recovery of the original data, even in absence of several original measurements. Our proposed method, thanks to a proper design of a delay function, orders the transmission of these measurements, being the nodes farther from the fusion center, the ones starting this transmission. This way, intermediate nodes are more likely to introduce their measurements in a packet traversing the network and to apply the CS technology. This way the contribution is twofold, adding different measurements to traversing packets, we reduce the total overload of the network, and also reducing the size of the packets thanks to the applied compression technology. We evaluate our solution by using ns-2 simulations in a realistic vehicular environment generated by SUMO, a well-known traffic simulator tool in the Vehicular Network domain. Our simulations show that CS-VDH outperforms Delay-Bounded Vehicular Data Gathering (DB-VDG), a well-known protocol for data gathering in vehicular sensor networks which considers a specific delay bound. We also evaluated the proper design of our delay function, as well as the accuracy in the reconstruction of the original data. Regarding this latter topic, our experiments proved that our proposed solution can recover sampled data with little error while still reducing the amount of information traveling through the vehicular network.

## 1. Introduction

Vehicular ad-hoc networks (VANETs) have drawn the attention of both the industry and research communities due to the number of services and applications they can provide in real life to different entities such as governments, car manufacturers, and communication operators. In the last few years, a new application domain called Vehicular Sensor Networks (VSNs) has emerged. It comes from the integration of wireless sensor networks (WSNs) and VANETs. That is, instead of deploying a high number of sensors in a specific urban area to measure a particular data of interest, cars equipped with sensors can be used as mobile sensing nodes. Moreover, vehicles are also responsible for forwarding the gathered information to the fusion center (FC) which is the destination of all the sensed data and the place where data analytics take place.

The issue of gathering information in an ad-hoc network is not a novel topic and has already been widely studied in the wireless sensor network (WSN) literature. Nevertheless, comparing WSNs and VSNs, there are two main differences between them that prevent the same techniques already proposed for the WSN domain to be applied to the VSN one. The first of these differences is the high mobility of the VSN nodes. Whereas in WSNs, sensors are deployed at specific and fixed positions within a region of interest, in VSNs, vehicles are continuously moving with high speed along streets and roads of urban scenarios. Additionally, the presence of traffic signs and traffic lights cause partitions in the network, the creation of platoons of vehicles, with the corresponding high variability of the wireless links among them. The second difference is related to the requirements the nodes have in terms of performance and energy consumption. Nodes in a WSN are usually equipped with short-spanned batteries and constrained processing and storing capabilities. This means that these different scenarios do not impose the same restrictions and conditions when designing communication solutions.

Traditionally, data harvesting protocols take advantage of intermediate nodes to append new information into traversing packets as they forward them to their corresponding destinations reducing this way the number of issued messages, but increasing the packet size as new information is included. Nevertheless, a collection of sampling methods in Information Theory known as compressed sensing (CS), compressive sensing, compressive sampling, or sparse recovery has emerged during the last years [1]. Its key aspect is the idea of reconstructing the original information of a signal using only a few samples without losing accuracy in the reconstruction process. Additionally, CS is able to obtain an accurate approximation of the data by using a small number of generalized measurements, which are known as projections. We explain the essential operation of CS in Section 2.

Considering the specific requirements and conditions of VSNs, this paper proposes a data gathering solution which comprises two stages: (i) a query dissemination process where the FC broadcasts a message within a determined region of interest, and (ii) the harvesting stage where vehicles send their measurements back to the FC in an efficient way. In our case, we consider the need of relying in a VANET routing protocol for efficient transmission of the packets, and the reduction of the payload size of the data packets compared to the case of gathering all the sampled data. For this purpose, a delay function controls the time at which nodes start sending their sampled data. This function is designed in such a way that the transmission of the information is started by the nodes farther from the FC, and intermediate nodes take advantage of the received packets to append their sampled data as the data packet is forwarded. Finally, thanks to the use of CS technology, the new data is combined with the previously stored information without practically increasing the overall size of the packet.

The remainder of this paper is organized as follows. Section 2 provides an overview of the operation of the CS technology. A review of the main related work is presented in Section 3. In Section 4, a thorough view of our proposal is described, detailing every phase of the harvesting protocol. The evaluation of our proposal by means of simulations is presented in Section 5. Finally, Section 6 concludes the paper.

## 2. Background

The Compressed Sensing technology was proposed by Candès and Donoho among others [1,2,3] as an alternative to the traditional sampling methods following the Nyquist–Shannon theorem. Later on, Candès and Wakin [4] presented a straightforward introduction to the CS technology. In this paper, the authors motivate the use of this technology, also describing its primary purpose, capturing the salient information of a particular signal of interest efficiently.

Traditional sampling methods require to sample a signal at least at a frequency of twice the signal bandwidth (Nyquist–Shannon sampling theorem). However, the argument posed by CS is that regarding signal information, all the elements of the signal are not always significant.

For instance, if we ask somebody for a random number, it will give it in base 10 by default. For instance, 11, this base is called in signal context the acquisition basis or sensing basis, but we can change its representation using a different base like hexadecimal, obtaining a different value, *b*. Therefore, we have selected the hexadecimal basis as its representation basis. Although we have two different values, 11 and *b*, both of them correspond to the same number; however, *b* has only one digit while 11 has two of them.

**Figure 1:**
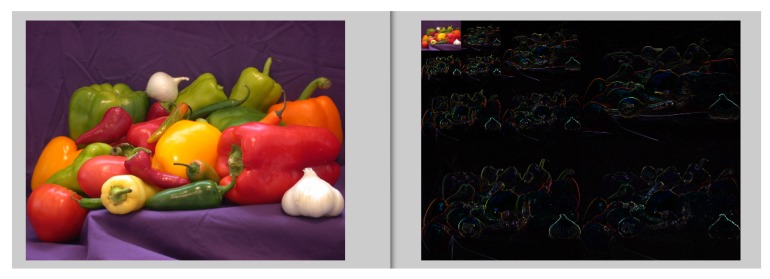
An image as example of a signal represented in traditional and wavelet basis.

The same happens to the signal representation. We can obtain a signal corresponding to an image whose components are the pixels and their colors, or we can use a different basis to represent it, lowering the amount of information. For instance, Figure 1 presents a wavelet transform of an image where the black pixels of the wavelet transform do not contain information. This basis uses, therefore, fewer data to represent and store the image compared with the traditional pixel and color representation. Its advantage is that we can recover the original image in the case is needed without hardly losing accuracy.

CS asserts it is possible to recover specific signals from far fewer samples than traditional methods use.

In order to do it, CS relies on two principles: sparsity and incoherence:

- A signal is sparse when the information rate is less than the signal bandwidth [1,2], i.e., only a few of the signal components are relevant, being the majority of them dispensable. For instance, if the majority of the signal components have the same values, and only a few of them have a different one, as we illustrated in the wavelet transform of the image in Figure 1. Transmitting only the relevant components in a compressed way makes it possible to reconstruct the original signal by the receiver while saving network resources.
- Incoherence, on the other hand, measures the correlation between the representation, also called sparsifying basis, and the measurement one. Thus if a signal is sensed using a basis Φ while for its representation, it is used another basis Ψ. The less correlation between the elements of both bases, the more incoherence both bases will have. Samples must also be incoherent. Taking two samples, the more correlated they are, the less information will be provided in global terms because we receive two times the same value.

Fulfilling the above principles of sparsity and incoherence, CS states that an original signal *x* of dimension *n* can be compressed by selecting *k* random measurements, k<<n, in the following way:

(1) $$\begin{array}{c} \left(\begin{array}{c} y_{1}\\ y_{2}\\ \ldots \\ y_{k} \end{array}\right)=\left(\begin{array}{c} a_{11}a_{12}\ldots a_{1n}\\ a_{21}a_{22}\ldots a_{2n}\\ \ldots \\ a_{k1}a_{k2}\ldots a_{kn} \end{array}\right)*\left(\begin{array}{c} x_{1}\\ x_{2}\\ \ldots \\ x_{k}\\ \ldots \\ x_{k+1}\\ \ldots \\ x_{n} \end{array}\right)\Rightarrow \\ \Rightarrow y=\left(\begin{array}{c} a_{1}\\ a_{2}\\ \ldots \\ a_{k} \end{array}\right)x\Rightarrow y=Ax, \end{array}$$

where ***y*** is the resulting compressed vector, ***A*** is the representation basis where the signal must be sparse, and ***x*** is the original signal also expressed as a vector. That is, the components of the signal *x* are projected onto the representation matrix that is used to transmit the information expressed as the elements of the vector *y* in a compressed manner, i.e., each component of *y* is represented as $y_{k}=<a_{k},x>$.

CS guarantees that, with this vector ***y*** with far fewer elements, *k*, than the dimension of the original signal, *n*, i.e., k<<n, it is possible to recover with high accuracy the original signal ***x***.

Let us explain this projection operation with an example. Given a signal represented as a vector $x^{T}=\left(x_{1},x_{2},x_{3},x_{4}\right)$, and a representation basis $A=\left(6,2,4,5\right)$ which for this example contains only one row. The projection is carried out like the next equation details.

(2) $$y=Ax=\left(\begin{array}{cccc} 6 & 2 & 4 & 5 \end{array}\right)\left(\begin{array}{c} x_{1}\\ x_{2}\\ x_{3}\\ x_{4} \end{array}\right)=6x_{1}+2x_{2}+4x_{3}+5x_{4}=Z.$$

So basically we have projected the vector ***x*** onto the projection basis ***A*** obtaining the projected value ***y = Z***. It is worth mentioning that initially, we had a signal with three components $x_{1},x_{2}$, $x_{3}$ and $x_{4}$ while the projected value comprises only one component *Z*, reducing therefore the amount of information.

**Figure 2:**
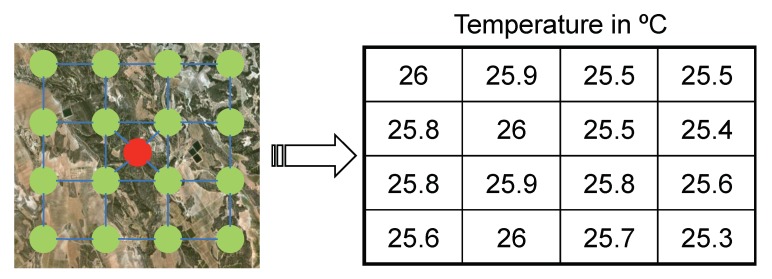
Wireless sensor network (WSN) measurements viewed as the components of a signal represented by a matrix.

The application of this technique to WSNs is described in [5], where the measurements obtained by each sensor of the network are seen as the components of a signal as Figure 2 depicts, applying CS to compress this information and its subsequent recovery.

Therefore, knowing the appropriate basis where this signal is sparse, nodes can apply CS generating projection vectors that compress the information as it is sent to the FC without a significant increase in the size of the packet.

Until now, we have explained the compression process of the CS technology but we have not dealt with the reconstruction process. This consists in the following minimization of the $l_{1}$ norm [4]:

(3) $$\min_{\tilde{x}\in R^{n}}{\left|\left|\tilde{x}\right|\right|}_{l_{1}}\mathrm{subject}\;\mathrm{to}\;\Phi \tilde{x}=y,$$

where ${\left|\left|.\right|\right|}_{l_{1}}$ represents the $l_{1}$ norm of a vector that is $\sum |x_{i}|$.

The reason for minimizing the $l_{1}$ norm of the vector, instead of minimizing the $l_{0}$ was proposed by Candès and Tao [6] because of being a good convex approximation to the latter minimization problem.

Although the target of this paper is not the analysis of the different techniques to recover the original signal *x*, i.e., the minimization of the $l_{1}$ norm of the vector, there are different research works which have successfully addressed this optimization problem. They are, a greedy algorithm called Matching Pursuit [7], as well as different improvements like Orthogonal Matching Pursuit [8] or Fast Bayesian Matching Pursuit [9], another statistical approach named Bayesian Compressive Sensing [10], and a convex optimization approach [11].

## 3. Related Works

As commented at the beginning of the paper, our principal interest is the definition of an efficient strategy to harvest the information of a determined urban region of interest (RoI) using the vehicles’ sensing equipment. Regarding this task, a survey of urban vehicular sensing platforms is provided by Lee et al. [12], where they analyze protocols such as Mobeyes [13], FleaNet [14] and Vehicular Information Transport Protocol (VITP) [15]. They are described in the following paragraph, highlighting the reason they do not meet the specific requirements of the VSNs.

Mobeyes is a proactive urban monitoring strategy where every node that performs a measurement sends a packet with a summary of its recently sampled information to its neighbors. In addition to the sensor readings, these summaries include relevant information like the timestamp and its location. In conventional sensor networks, this information is dispatched to the nodes responsible for analyzing the sampled data. However, in VSNs, this mechanism is not practical due to the vast amount of generated data.

Besides, Mobeyes also allows an on-demand harvesting strategy (Mobeyes-ODH) that works as follows. First of all, the fusion center issues a query request which is broadcast in a region of interest. The receiving nodes immediately answer to this query with its data. So, such a scheme has the issue of transmitting a lot of packets that overload the network.

In FleaNet query dissemination protocol, queries are advertised only to 1-hop neighbors. After receiving the query, neighbors store the query without any further forwarding. This way, queries are only spread by means of vehicle motion. FleaNet also provides a mechanism to deliver data using multihop communications, but as in Mobeyes, the early response of the nodes receiving the request message overloads the network.

VITP, on its side, allows nodes to aggregate information and report summarized results to the requester. This aggregation consists of piggybacking partial results, so although they reduce the number of packets, they still increase their size with each new partial result added to the packet.

Another interesting solution is proposed by Palazzi et al. [16], called Delay-Bounded Vehicular Data Gathering (DB-VDG). This protocol follows the same strategy as we propose in this paper with two variants (Speed-Based Strategy Selection method) SBSS and Distance-Based Strategy Selection method (DBSS). Data harvesting is divided into two stages. In the first stage, the FC node broadcasts a query message in a specific urban region. In the second one, nodes equipped with sensors that receive this query send back the sensed information taking into account the lifetime of the received query. Depending on the selection method, intermediate nodes decide to forward the messages or being themselves ferries for the data. SBSS takes into account the velocity of the node in order to make this decision, while DBSS simply selects the strategy based on the distance to the FC. In both cases, when forwarding is applied intermediate nodes append multiple samples into the same packet preventing the overhead to excessively increase by flooding a large number of (smaller) packets in the network.

All in all, the above proposed protocols do not use data compression, a key aspect that we consider pretty important. They only take advantage of traversing packets to append new information as they encounter a new intermediate node. Therefore the size of these packets depends on the number of intermediate nodes forwarding the packet or the areas of the region of interest that the packet must traverse to reach the destination. Nevertheless, with an efficient gathering method, and compression technique we can benefit from this circumstance to, firstly, optimize the number of packets traversing the network, and secondly compress the payload of the packets, with the consequent reduction in the network overload.

The topic of gathering information has been widely studied in other research fields like WSNs, where the specific restrictions of the sensors regarding processing, memory and battery have led to efficient harvesting approaches in relation with the previous factors which have also considered compression techniques for a reduction of the information to be transmitted. Among the different compressing techniques that have been applied [17,18,19,20], CS emerged with much strength due to its high compression rate and the accurate reconstruction with respect to the original data.

Haupt et al. [5] provide a review of CS, as well as its application to different fields like WSNs, where the compression of the information can be even applied to the transmitted information directly in the air as proposed Bajwa et al. [21], or Feizi et al. [22]. The former presents a technique called compressive wireless sensing which allows the combination of two signals. The latter proposed another scheme called sparse distributed compression which avoids the energy consumption in combining the sensing information within each node.

Although these techniques are out of the scope of our research domain, there are other approaches applied to the networked data by using projection vectors to compress the information that is more suitable for our research area. The underlying idea is to define a RoI of a specific scenario which is divided into *r* rows and *c* columns. This way we obtain an r×c matrix, whose cells contain a value corresponding to the sensor reading inside the corresponding subdivision of the RoI. The matrix is now seeing as a signal where the CS technology can be applied.

Works like Chou et al. [23] apply the CS technology in the WSN domain. In this paper, the authors propose an adaptive scheme managed by the FC. In a first step, nodes randomly decide to send their measurements without any compression scheme to the FC. After receiving this information, the FC chooses a projection vector to obtain information about the areas where it has vague or imprecise information. This message, as it is transmitted through the network, is used by the sensor nodes selected by the projection vector to incorporate their sensed information. Eventually, the projection vector is fulfilled and sent back towards the FC.

Additionally, Zhang et al. [24] present an adaptive compressed sensing algorithm for wireless image sensor networks where higher sampling rates are assigned to blocks that are less compressible. Zhou et al. [25] propose a gathering scheme for WSNs based on the CS theory over graphs. Another work from Pacharaney et al. [26] introduces the concept of clusters and CS over WSNs. Other works, such as Valsesia et al. [27] computes CS projection in networks by means of randomized aggregations of signal values in a neighborhood.

Unlike in WSNs, nodes in VSNs move inside an urban scenario. Therefore, although compressing techniques in the network were applied, the FC will not have enough information about where these measurements have been taken. This is the reason why we do not apply compression in the network, but in at data level using the projection vectors already mentioned.

On the other hand, CS has been also applied to the VSNs. Yu et al. [28] present a theoretical work where nodes gather information at intersections, and after the acquisition of several data, they apply CS to these samples that are then transmitted to the FC.

More recently, Huang et al. [29] proposed a complete framework for acquiring information which can adjust the parameters of the measurement matrix in CS according to the characteristics of sensed target data and the distribution of vehicles, keeping this way the sparsity feature required for a proper application of the CS technology. Moreover, vehicles act as ferries collecting data along their trajectory. Finally, Lin et al. [30] introduced a compressive sensing approach for capturing and recovering data from a VSN. Nevertheless, they do not mention the protocols used for distributing the information from vehicles to the fusion center and only apply the CS technology from a theoretical point of view.

Table 1 summarizes the different features extracted from the different proposed solutions where data gathering has been addressed, either by using compressing techniques or not, or even if they come from the WSN or the VSN domain. As the reader can observe, data gathering proposals which apply CS are mostly focused on the adoption of this technology without considering other aspects such as the distribution of the query in a region of interest, or if an underlying routing protocol is required in order to make the transmission of the sensed information to the FC more efficient.

In this paper, our objective is to analyze through network simulations the impact of CS in a VSN environment considering the high mobility of the vehicles, their mobility restrictions following roads, and link breakage and variability these factors cause in the network. We have considered also the necessity of efficiently broadcast a query message to a specific RoI, as well as the transmission of the information from the nodes inside the RoI to the FC. For this purpose, unlike the related works commented above, we have defined an efficient harvesting scheme called CS-Vehicular Data Harvesting (VDH) which includes a geocast solution for an efficient query distribution in a RoI, a VANET routing protocol for efficiently receiving the information from the vehicles, and the integration of such a routing protocol with the CS technology.

## 4. CS-VDH

**Figure 3:**
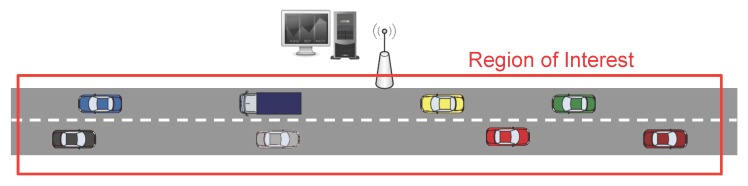
Example of an RoI.

**Figure 4:**
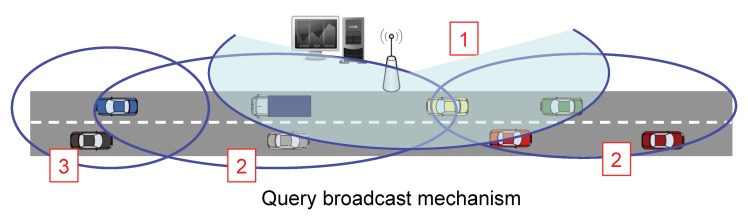
Query broadcast stage 2.

With the goal of taking advantage of the CS technology to improve the efficiency in data gathering protocols for the particular domain of VSNs, we propose a solution called Compressed Sensing based Vehicular Data Harvesting (CS-VDH) which comprises two main stages:

In the first stage, as Figure 3 and Figure 4 show, the Fusion Center (FC) issues a query message (CSQuery) with the intention of gathering information like temperature, humidity or congestion, to name different alternatives. This message is intended to be broadcast within a specific region of interest (RoI). So, although for simplicity in our figure, we have established the FC close to the RoI, this distance can be extended to several kilometers requiring a hop by hop transmission to reach the RoI. Eventually, this message is broadcast to all the vehicles inside the RoI.

**Figure 5:**
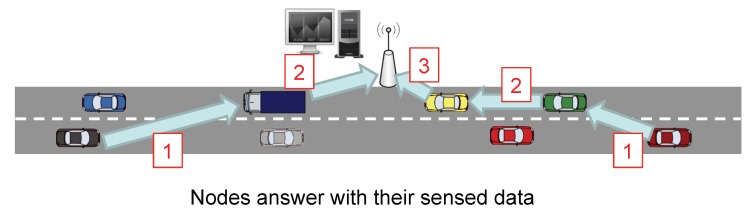
Harvesting stage.

The second stage is the gathering process. Its aim is that of lowering the overload of the network by reducing the number of transmitted packets as well as their size. So, as Figure 5 depicts, the vehicles located farther from the FC are the first ones in sending their sensed information. Intermediate nodes take advantage of these data packets they have to forward to include their measured information. Rather than appending new raw data, nodes use CS to combine the projections of the data so that extra information is incorporated into the data packet.

**Figure 6:**
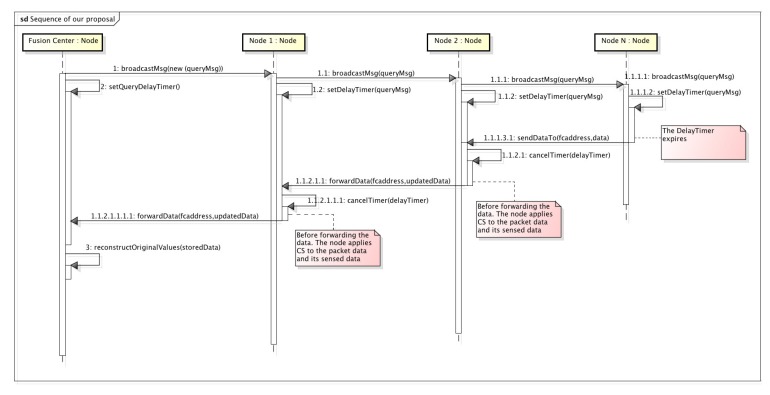
CS-Vehicular Data Harvesting (VDH) sequence diagram showing the query distribution and harvesting stages.

Figure 6 provides a sequence diagram giving a complete view of the messages transmitted in our proposal, as well as the processing activities that nodes must also fulfill to answer to the FC with the information they have sensed.

The process is triggered by the FC which broadcasts a query message (CSQuery) containing the address of the FC, as well as its location to a specific RoI. This process relies on a geocast routing for a proper dissemination of the message. In our example, the CSQuery is forwarded by Node1 and Node2. Each node, after receiving the query message, sets up a delay timer which represents the time nodes must wait before generating a new data packet with its sensed information. This timer is calculated based on the distance from the receiving node to the FC. To do so, vehicles and the FC are assumed to be equipped with a global positioning system. Later, we give a thorough explanation of this delay in the following sections.

When the delay timer of Node N expires, i.e., the node farther from the FC, it starts sending its sensed information to the FC. To do so, it generates a packet containing the gathered data and a boolean vector. This vector represents the areas by which we have divided our RoI which was also included in the query message by two opposite corners. So, once a sensor reading corresponding to a specific area of the RoI is gathered, the corresponding boolean vector element is marked as true. When an intermediate node receives the data packet, it cancels the delay timer in case it has not expired yet, and combines its measured data with the one contained in the data packet. That combination of data is performed using CS, as explained in Section 2. That is, taking Figure 2, as well as the Equation (2) as the reference, when a node takes a measurement which corresponds to the first cell. It multiplies this value with the values of the representation matrix. $proj_{1}=26\times x_{1}$. This packet is then transmitted, and when it reaches a node moving inside a different cell of this matrix, for instance, the cell (1,2), it now projects its value in the following way: $proj_{2}=proj_{1}+25.9\times x_{2}$. This operation is repeated until it reaches the FC.

On the other hand, when the timer of a node expires, it generates, as Node N did, a new data packet including only its own sensed information. Again, this packet will be sent to the FC passing through the intermediate nodes, which will insert their information, as previously commented.

### 4.1. Design Issues

Given the complexity of the VSN scenario to broadcast query messages, as well as to send measurements back to the FC, we consider that there exist several design issues worth highlighting in this paper. Starting with the distribution of the query message we enumerate the required fields to be transmitted:

- A sequence number to identify the packet.
- The FC address.
- The FC location.
- The RoI, specified by the location of two opposite corners.
- The projection vector.
- The number of cells per row.

The three four-fields are required for the underlying geocast routing protocol for proper dissemination of the message inside an RoI geographically delimited by the two opposite corners of a rectangle and avoiding the retransmission of duplicated packets thanks to the sequence number. Finally, the two latter fields correspond to the projection vector which is a single row vector related to the representation matrix, and the number of cells per row which helps nodes to map the representation matrix containing a number of cells per row to the single row vector. This is further explained below.

**Figure 7:**
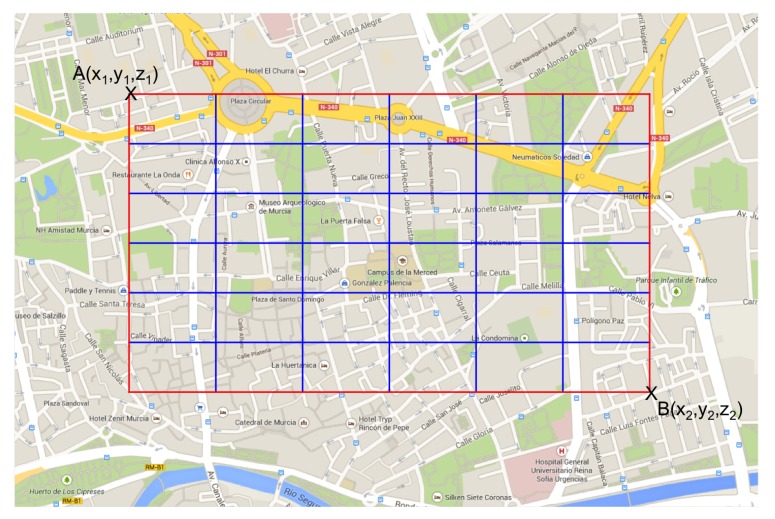
Definition of a RoI with 6×6 cells.

As commented in Section 2, the application of CS is based on the multiplication of two vectors of the same size. Since one of the vectors, the projection vector, must be sent within the CSQuery, we have to set its size a priori. For this reason, we decided to divide the RoI into cells, as Figure 7 shows. This way, we can map node locations to the cells of the RoI. This approach provides an extra advantage. If a node receives a data packet from another node and it already has the information of the cell where the node has taken its measurement, which is easily checked by looking at the boolean vector, it only forwards the packet without including new information.

**Figure 8:**
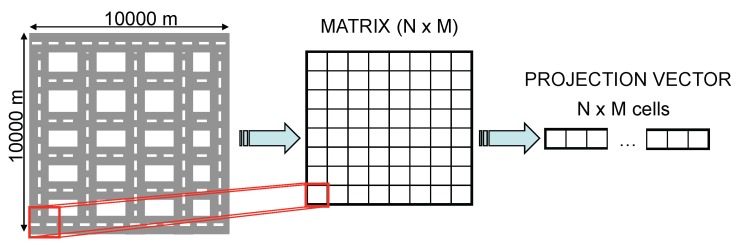
Matching process to obtain a projection vector.

Another interesting design issue is the adaptation of the RoI matrix to a vector since CS operates with vectors. To do so, each of the rows of the matrix are concatenated, obtaining a single row vector with all the cells of the matrix as shown in Figure 8. Finally, we insert in the CSQuery message the size of the rows of the original RoI matrix, so that nodes could reconstruct the original RoI.

With this last change, the operation is like in the previous CS example allowing us to compress the information of various locations, as exemplified in the following equation.

(4) $$\begin{array}{c} y=Ax=\left(\begin{array}{cc} 6 & 2\\ 4 & 1 \end{array}\right)\left(\begin{array}{cc} x_{1} & x_{2}\\ x_{3} & x_{4} \end{array}\right)\Rightarrow \left(\begin{array}{cccc} 6 & 2 & 4 & 1 \end{array}\right)\left(\begin{array}{c} x_{1}\\ x_{2}\\ x_{3}\\ x_{4} \end{array}\right)\\ =6x_{1}+2x_{2}+4x_{3}+1x_{4}=U. \end{array}$$

One of the significant advantages that CS provides is that we can combine information at different moments. For instance, assuming the FC has already broadcast a query message in a RoI containing the projection vector (6,2,4,1), if one node, for instance, has the measurement of position (1,1), it can project its value obtaining $6\times x_{1}$ and transmit it to the following intermediate node. Moreover, the intermediate node can also combine its sensed information, for instance, of position (2,2) just by adding its projected value $1\times x_{4}$ resulting in a new value $T=6\times x_{1}+1\times x_{4}$. This suppose a notable compression rate compared to transmitting the two values in the same packet.

This is possible because, according to CS, a node can combine its sensed data if the cells to which the data refers has not been included yet. For this reason, a packet must include information about which cells contributed information to the carried data. In order to deal with this problem, we introduce a boolean vector indicating which cells provided information to the combined data. This vector has the same size as the number of cells in the RoI.

Based on the boolean vector, a node can quickly check if its sensed information can be combined with that of a received data packet. This process is done simply by checking that the cells with a ‘1’ in its own boolean matrix are set to ‘0’ in the received boolean matrix. Otherwise, the combination cannot be made. When a node combines data, it updates the cells of the resulting boolean vector accordingly. For instance, let us assume that we have defined a 2×2 RoI. That nodeA has added the information of the cells (1,1) and (1,2) represented as the following equation describes.

(5) $$\begin{array}{c} Booleanvector=\left(\begin{array}{cc} 1 & 1\\ 0 & 0 \end{array}\right)\Rightarrow \left(\begin{array}{cccc} 1 & 1 & 0 & 0 \end{array}\right). \end{array}$$

If nodeB intends to insert the information of the cell (1,1), just by checking that it is marked as true, it knows that the information has already been added, and directly forwards the message to the next hop.

After identifying the main obstacles that our proposed solution must overcome, as well as a specific mechanism that can help us in overcoming them. In the next subsections, we shed some light on the detailed operation of our proposed solution.

### 4.2. Query Distribution

In this first phase, a query message (CSQuery) must be distributed inside a precise RoI so that the mobile nodes inside this area could retrieve the sensed information requested by the FC. For this purpose, we must employ a low-level protocol to flood such a query within the RoI. This task is a research topic that has already been investigated in the VANET domain and whose proposed solutions receive the name of geocast routing protocols. Protocols such as PIVCA [31] or AckPBSM [32] have presented outstanding results in this area.

Our solution for data gathering in VSNs is agnostic to the relying technology responsible for this task which can be easily adopted. Nevertheless, it does consider the design of the information representation that must be carried inside the CSQuery message to be transmitted. The different elements that must be included in these messages were already enumerated in the previous section.

The sequence number is an identifier associated to the packet and its issuer which is usually used for detecting duplicated packets, ignoring them this way.

The address and location of the FC are required since it is the destination of all the sensed information and all the mobile nodes providing their information or forwarding it must be aware of. The number of cells is also essential for the vector-to-matrix conversion. Finally, the most significant component needed to apply CS is the projection vector, which allows mobile nodes to combine and compress their information by projecting their sensed measurements. In Section 2 we explained that CS relies on two principles to its correct operation, sparsity and incoherence. The projection vector must transform the sensed measurements, changing its representation in a sparse basis where only a few components are relevant.

The main shortcoming is that there is not a solely one representation basis that makes all kind of measurements to change their representation to a sparse domain. By contrast, this representation basis, and therefore the projection vector, must be chosen according to the type of measurements that mobile nodes have to provide: temperature, $CO_{2}$ concentration, the average speed of an urban sector or humidity to name a few. As we will detail in the evaluation section, we have experimented with the temperature information of the city. This type of measurement has specific properties such as that it does not drastically vary (i.e., high variances in a couple of seconds), and that its new values are related to the ones of a previous moment. For this reason, we have opted to specify a representation matrix based on the variance of these values in a previous moment.

### 4.3. Harvesting Process

Finally, the harvesting process is the phase where CS takes place to compress the information as it traverses the network. In Section 3 we have reviewed different proposals applied to VSN which actually makes use of the CS technology. However, they are focused only on the application of the CS technology using vehicles only as ferries to include their sensed information as they follow a path [29], or they do not provide relevant information about how this information must be transmitted in an efficient way [30].

Our proposed solution considers that the information sensed by the mobile nodes inside a RoI may expire. For this reason, an efficient protocol for transmitting the sensed information must be employed. There are many VANET routing protocols which are specialized in this task such as: GPCR [33], GPSR [34], SAR [35], GeOpps [36] or BRAVE [37] to name a few.

On the other hand, the content of the data packet is also relevant for the application of CS and its later recovery process. Each data packet contains the projection vector, the number of cells per row in this vector, and a boolean vector with the same number of items as the projection vector transmitted in the query message. This boolean vector indicates the areas whose values are already included in the data packet. That is, the first node in generating its data packet sends its sensed information along with the boolean vector. This latter is set according to the areas where the measurements were taken. When an intermediate node receives this packet, it checks whether its sensed information can be included in the packet by looking at the non-marked cells of the boolean vector. If the answer is positive it can apply CS to the packet inserting its information. Finally, it marks the cells of the boolean vector corresponding to the appended information.

In our proposal, we have defined two different operation modes for the harvesting stage. In the first one, when the delay timer expires, or a data packet has been received by a node, it inserts its sensed data at its current location. In the other operation mode, the node does not only insert its measurement in its current position but also those taken in other previous locations.

In the first operation mode, only a single measurement can be added by each intermediate node inside the RoI, following the method explained above. If there already exists a measurement corresponding to the area of the RoI to which the intermediate node could contribute, it only forwards the message.

In the second one, the process is a bit more complicated because the node that receives the packet may also have compressed information to be sent. In this case, the node must check if the two boolean vectors are compatible, i.e., they do not overlap,. This happens when both vectors are disjoint, i.e., when they do not share any values. Let us explain it better with a couple of examples:

Let us assume that the information of two nodes are the ones indicated in Table 2. Node 1 has sent a message with the information of the areas 1 and 2. That is, the projection P1 and the boolean vector (1, 1, 0, 0) corresponding to the first row of the table. This message is received in its way to its destination by Node 2, who has already stored the information of the areas 3 and 5, so obtaining the projection P2 and the vector (0, 0, 1, 1) (the second row of the table). Since their information is compatible, the combination of both data is possible combining the information as the last row indicates.

However, in Table 3, the information of both nodes is incompatible. The projections cannot be combined because both sets of areas are not disjoint because both have inserted the value of Area 3, and if we combine them, we will introduce twice the term C∗$X_{3}$ in the compression form which is not correct from the point of view of CS. In this case, Node 2 will forward Node 1’s message generating a new data message with its information too.

### 4.4. Enhancement to the Basic Scheme

If every node inside the RoI transmits its sensed information at the same time, the network will be notably overloaded, and the data communication will be jeopardized in such a way that only a few data will arrive in the FC due to the contention, lack of resources, and the likes. Examples such as Mobeyes, and Mobeyes-ODH, were presented in Section 3 which have these problems because nodes immediately respond after receiving the query sent by the FC.

This undesired behavior can be mitigated by sorting the different responses in such a way that, instead of having all the nodes sending their sensed information at the same time, they do it gradually. Thus, intermediate nodes can take advantage of traversing packets to introduce their sensed information in them. If the nodes farther from the FC are the first ones in answering with their sensed information, intermediate nodes are more likely to include their information as it is forwarded back to the FC.

For our proposal we studied the use of a delay function expressed by the following equation:

(6) $$y=p_{1}*e^{-e^{\frac{x-p_{2}}{p_{3}}}},$$

and whose parameters are $p_{1},p_{2},$ and $p_{3}$. We have used this function because of its flexibility. That is, by varying the values of its parameters, the function can change their shape.

Each of the parameters has a purpose in the function:

- $p_{1}$: this parameter limits the upper asymptote, that is, the maximum time (TMAX) a node must wait until it sends back its sensed information.
- $p_{2}$: it models the distance from which the answer time decreases exponentially.
- $p_{3}$: finally, $p_{3}$ models the number of points the slope has. The more points, the less steep the slope will be.

**Figure 9:**
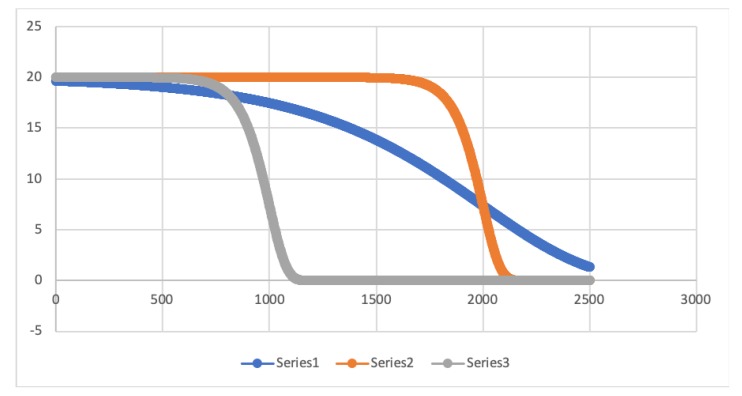
Representation of three different shapes the proposed delay function can have according to the values for $p_{1}$, $p_{2}$ and $p_{3}$ specified in Table 4.

Considering an urban scenario like the one of the city of Murcia with 4∗5 $km^{2}$ we made a first analysis of the selected mathematical function by varying its different parameters. Actually, we have used three different sets of parameters that change the response time that the mobile nodes have to depend on their distance as presented in Figure 9 and whose values can be seen in Table 4.

**Figure 10:**
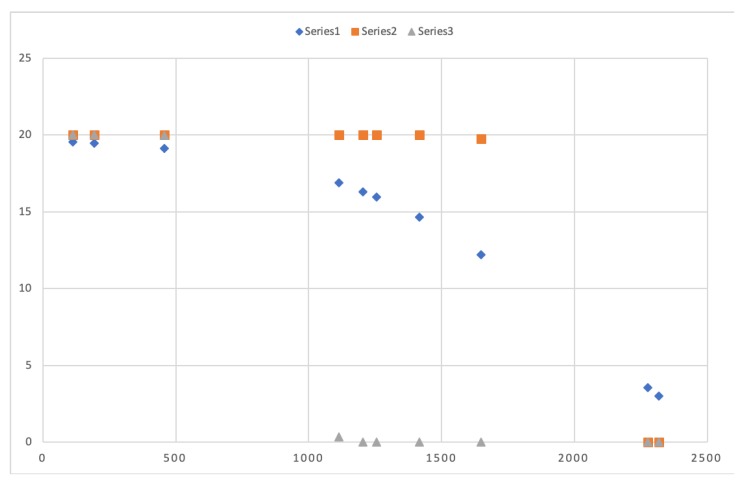
Delay function values for 10 randomly selected points with different distance to the FC.

Now let us select a set of 10 random points that correspond to mobile nodes positions with different distance to the FC which is placed in the center of the scenario. As we can see in both Table 5 and Figure 10, these positions obtain different delay time depending on the series. Series 1 values provide different delay time to different positions although they are nearby. Nevertheless, series 2 and series 3 provide a similar delay time to nodes located nearby each other. This is caused by the steep slope both series present in the figure having the majority of the represented points in both the maximum delay time, or 0 delay time.

Additionally, another conclusion that we can extract for the graph is that both series 2 and 3 do not provide a suitable delay time because they maintain either a really high value or a low one for the most of the randomly selected points due to the value specified in the parameter $p_{3}$.

## 5. Evaluation

Our proposed solution comprises the whole process of distributing the query inside an RoI and the transmission of the sensed information from the vehicles inside this region to the FC. Each of these tasks requires a different technology to undertake this dissemination. On the one hand, a geocast protocol is required to spread the query message inside the RoI, and, a routing protocol capable of dealing with the features of the VANETs is also needed that assures the delivery of the messages to its destination.

Among the existing geocast solutions, we have selected AckPBSM [32] for the query’s broadcast because it is an adaptive protocol, suitable for vehicular scenarios and which have been proved to be efficient regarding the network overload and reliable in vehicular networks.

On the other hand, for the harvesting stage, we have selected BRAVE [37], which has also obtained an excellent performance in terms of packet delivery ratio and which also provides support for Delay Tolerant-Networks (DTN) allowing nodes to carry themselves the packets until they find a suitable neighbor for their destination.

Finally, the last aspect that we must tune is the delay function used to set the time nodes must wait before generating a new data packet with its sensed information.

In the simulated scenario, we have specified an RoI of 4047×5047 $m^{2}$, and we have set our FC in the center of the scenario. So, we have tuned the following parameters: $p_{2}=2000$, which specifies the distance to point where the slope of the function is produced; and $p_{3}=80$, which specifies the steepness of the slope. So, after setting these parameters, we ended up having the following equation

(7) $$y=T_{MAX}\times e^{-e^{\frac{x-2000}{80}}},$$

which corresponds to the second sort of series in Figure 9.

TMAX, which corresponds to the $p_{1}$ parameter, is the value of the lifetime of the response. That is, the maximum waiting time the sensing nodes have to wait until they generate a packet with their measurements. For our simulations, we have set a TMAX value of 20 s.

### 5.1. Simulations

In the literature, we have found different proposals that applies CS to the VSN domain. Nevertheless, they analyze this aspect from a theoretical point of view or do only consider the compression aspect using the vehicles as ferries, without considering the mobility of the nodes and their effects on the vehicular network. Therefore, we have opted to compare our solution with another proposed solution called DB-VDG already introduced in Section 3 which also has presented interesting results from the point of view of efficiency and network overload.

**Figure 11:**
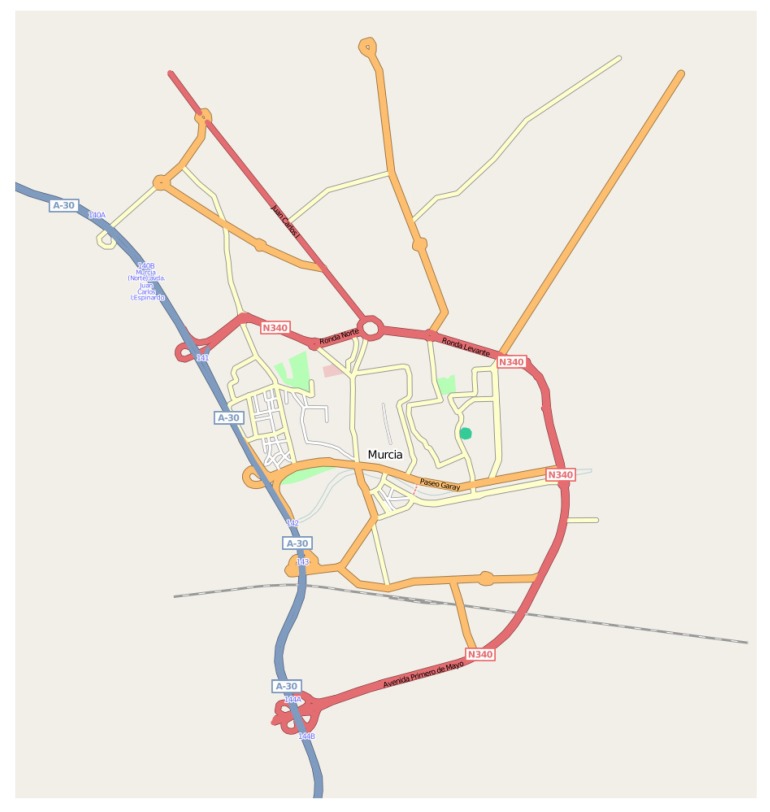
Urban scenario of the city of Murcia used in our simulations.

We have evaluated our proposal’s performance through simulations using the Network Simulator NS-2 (https://www.isi.edu/nsnam/ns/), version 2.33. For this purpose we have developed an urban scenario with the SUMO tool (http://sumo.sourceforge.net/) taken from the main streets and highways of the city of Murcia, Spain. Particularly, for our simulations, we have selected the most relevant streets, so our scenario consists of 53 streets and 28 junctions as shown in Figure 11. Vehicles move through 20 predefined routes at a maximum speed of 13.98 m/s (50 km/h) inside the city, and 22.22 m/s (80 km/h) on the highway that crosses the scenario during 445*s*. In this scenario, the following vehicles’ densities: 1/50,1/45,1/40,1/35,1/30,1/25,1/20,1/15,1/10 and 1/5 veh/route/s have been defined. This means that, for instance, for the less dense scenario which corresponds to the first value (1/50), in each of the defined route, a vehicle is injected in the scenario every 50 seconds. This means that, for a total of 10 routes defined for this scenario, the density of vehicles corresponds to the one described in Table 6.

Regarding the signal propagation model, we have used TwoRayGround for our simulations defining a coverage range of 250 m. Besides, we have run 10 different executions per scenario and vehicle’s density whose results are presented in graphs where 95% confidence interval are also included.

Since in the harvesting process there is only one FC, we have varied the random component seed, which is applied to timers making different vehicles to answer first in each execution of the simulation.

So, under the conditions specified above, we have evaluated the performance of our proposed solution performance against another gathering scheme, DB-VDG. Additionally, we compare variants of our proposed solution.

### 5.2. Comparison against DB-VDG

Comparing CS-VDH to DB-VDG as it was originally designed would be unfair because, in DB-VDG, every node at receiving a packet inserts its sensed information without taking into account the area in the map where the measurement was taken. That is, if different nodes receive a packet in the same area, they will append their information dramatically, increasing the packet size as intermediate nodes forward it to its destination. For this reason, we have adapted it to perform as our proposal does. That is, the urban RoI is divided into areas, and a node using DB-VDG only inserts the sensed measurements of its current area into the received packets, but only if this information is not already inserted in the packet.

After making these modifications to DB-VDG, we have compared both proposals as Table 7 presents. As we can observe, the overhead (in terms of number of messages) of DB-VDG in its two variants, SBSS and DBSS, is enormous. This tremendous overhead jeopardizes the performance of the network in terms of packet delivery complicating both query dissemination and harvesting tasks. The cause for such a behavior is heterogeneity and realism of the scenario, which makes DB-VDG generate a considerable number of messages.

On the other hand, our approach, in all its variants, obtains a better performance in both the query dissemination and the harvesting process overhead, which is about 200 times better. Besides, regarding the number of messages generated during the simulation, our compression proposal outperforms the one without compression, reducing the number of messages that are required. So, in light of these results, we can state that, although in reduced and controlled VANET environments, DB-VDG has provided promising results, in heterogeneous and realistic environments it encounter scalability problems causing a very high overhead in the network.

### 5.3. Impact of CS in the Overhead

We have proved that the improvement of CS-VDH compared with DB-VDG is notable. However, with this comparison, we do not have analyzed the benefits of the CS technology concerning the compression and size of the transmitted packets. For this purpose we have compared three variations of CS-VDH: two of them integrate CS, whereas, in the last one, intermediate nodes simply append their sensed information packet to be forwarded without applying any compression technique. On the other hand, the difference between the both CS variations consists of the two different operation modes, CS with and without accumulation, that we previously commented in Section 4. We refer to the first one as CSPure, to the second one as CSAccum and to the one without CS as NoComp.

**Figure 12:**
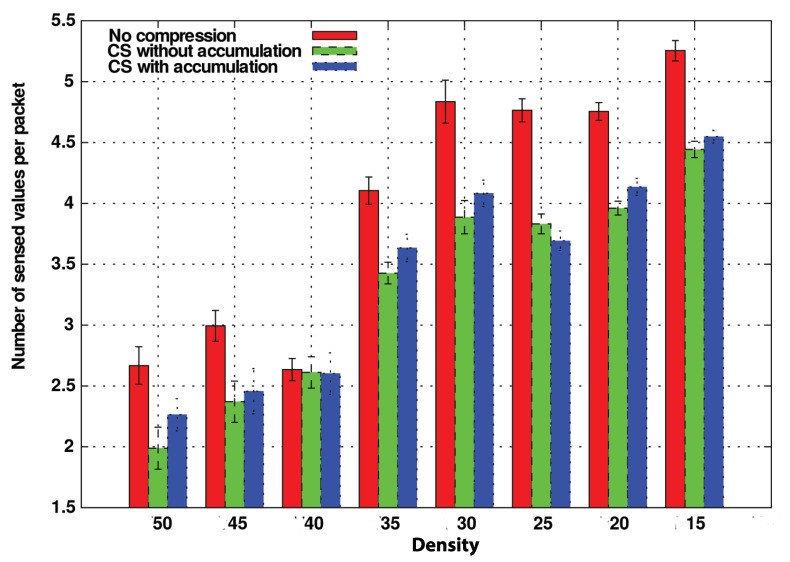
Number of sensed measurements carried in packets for CSAccum and CSPure for maximum time (TMAX) = 20 s. Density in x-axis corresponds to the name given to that density in Table 6.

In Figure 12, we measure the amount of sensed values that nodes can carry in these operation modes without taking into account the packet size.

We consider the NoComp option the best possible solution in terms of the number of delivered packets due to its straightforward operation mode because new information is appended directly into the packet in case it does not exist.

Regarding the compression variants, both CSPure and CSAccum take more time to comprise the information, being CSAccum the one with the most complex operation mode. Although by CSAccum, a node can insert more sensed values at once. The compatibility problem penalizes its performance. Despite that, its performance in terms of carried values is also good.

**Figure 13:**
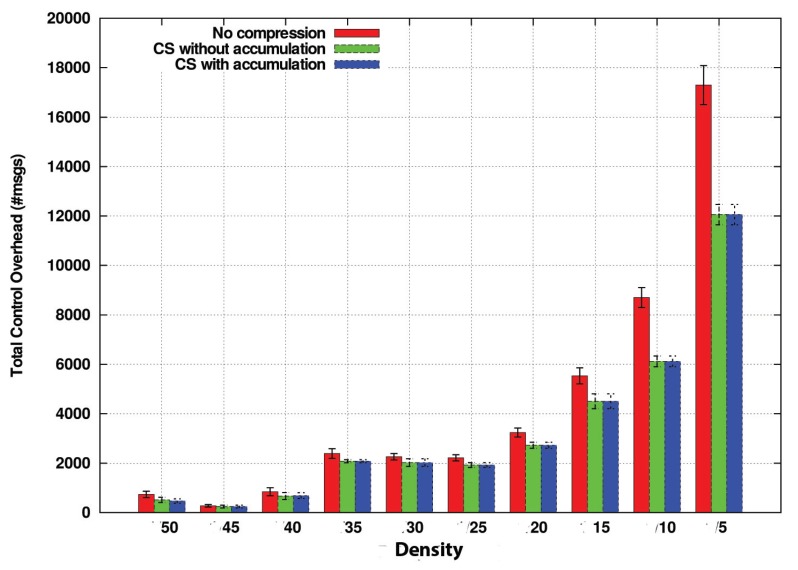
Overhead of compression strategy vs no-compression strategy in msgs for TMAX = 20 s.

In light of this graph, the NoComp approach can be considered as the ideal method, because it is the one which can carry more sensed values, improving the other CS options by 1 or 1.5 more carried values. Nevertheless, we still have not considered the overhead introduced in the whole network. Figure 13 compares this metric among the different options with respect to different vehicle densities. This way, now we are measuring the number of packets traversing the network.

As we can see, the benefits of CS concerning the overhead are more notable as the density of vehicles increases. The reason for this is that under lowly dense scenarios, for a distant node, the probability of finding a way to reach the FC is low. So, since the timers of nodes close to the FC will expire with high probability, this reduces the possibility of combining data in CS. Thus, the performance is similar to the NoComp approach.

On the other hand, in dense scenarios like 1/15, 1/10, and 1/5 veh/sec/route, the results are different. Nodes communicate more frequently, i.e., the likelihood of finding promising nodes that can forward data packets to the FC before their delay timers expire is higher. Therefore, they are more likely to combine their information and thereby to reduce the network overload.

Besides, overhead increases as the density does. This trend is less stronger in both CS strategies with a maximum overhead of about 12,000 messages for the highest density, whereas in the strategy without compression, the curve is more marked, reaching nearly 18,000 messages in the same scenario. This trend, therefore, confirms the better scalability of our approach in terms of control overhead.

### 5.4. Impact of the Maximum Waiting Time

The delay function specified in equation 6 governs the time nodes must wait for issuing a data packet to transmit their sensed information. This delay function has three parameters: $p_{1}$, $p_{2}$ and $p_{3}$.

$p_{1}$ corresponds to TMAX, the maximum waiting time for a node to send a new packet with its measurements. In this section, we make an analysis of this parameter which must be adjusted according to the lifetime of the gathered information. Specifically, we have studied the impact of TMAX parameter with the following values 2, 5, 7, 10, 20, 40, and 50 s.

**Figure 14:**
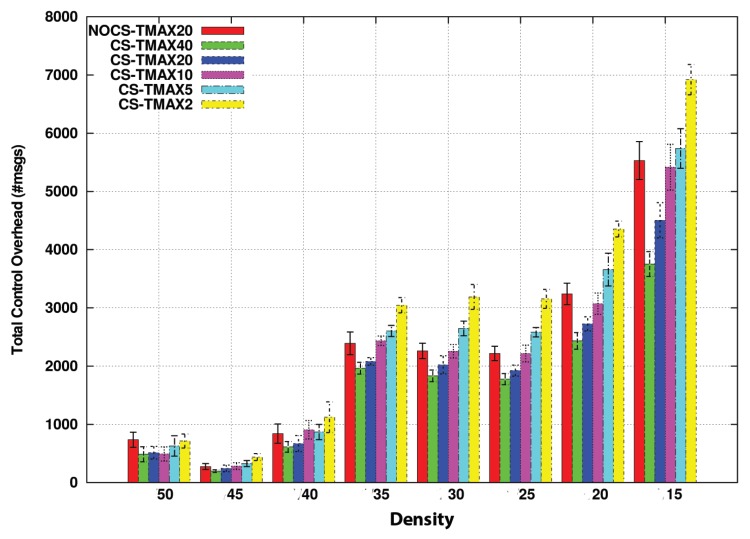
TMAX impact onto the overhead without accumulation.

Figure 14 presents the control overhead of both strategies with and without compression. We have obtained results for CS with different values of TMAX (2, 5, 10, 20, and 40 s), and we have compared it with the strategy without compression with a value of TMAX of 20 s.

A first conclusion that we can deduce from this graph is that CS outperforms the no-compression option when the TMAX value is higher than 20 s. Actually, a TMAX value of 10 s produces a similar performance to the no-compression strategy. This can be considered, therefore, a threshold value in this scenario.

Another satisfying conclusion we obtain from these graphs is that the advantages of using CS are more notable as vehicle density increases. In low-density scenarios, vehicles are less likely to find a neighboring node soon to forward the data to the destination. For this reason, their delay timer expires very often despite having a TMAX value of near one minute. However, as the density increases, the probability of finding a neighbor increases too. It does it in such a way that even a difference of a couple of seconds in TMAX causes a notable difference in performance, as we can see in the graph.

**Figure 15:**
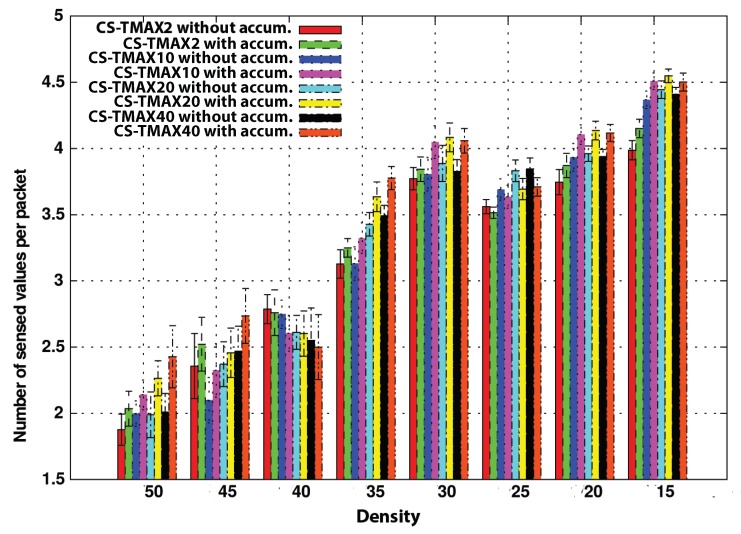
TMAX impact onto the carried data by nodes.

This also occurs when measuring the carried sensed values. Figure 15 again presents a rising trend as the TMAX values increases for every density. This trend reaches its maximum in highly dense scenarios where the maximum number of data is reached with a value of TMAX of 20 s, e.g., with density 30. We can figure out that there is a critical gap during the 10 first seconds, where the number of data presents more variate results.

Regarding the differences between using an accumulation strategy, given that the TMAX value increases, there is not a significant variation between using CS with or without accumulation. That is, it influences both strategies equally.

### 5.5. Reconstruction of the Data

In this paper, we have put our interest in analyzing the goodness of CS to consider it an excellent approach to harvest information in the scope of VSNs. This section provides an overview of the reconstruction process performed by CS without detailing its operations. We have used the Bayesian Compressive Sensing technique [10] to achieve the reconstruction, but there are other CS-based techniques already mentioned in Section 2 which provide similar results.

So, for the sake of this example, we have used the data collected of one of the harvesting operations from one execution of the simulations with density 15 (according to Table 6) in Matlab [38] to reconstruct the original data, that is the real values measured by the nodes, using the code of BCS using a relevant vector machine (RVM)(http://people.ee.duke.edu/ lcarin/BCS.html).

Initially, we set the temperature in the scenario according to a real temperature map of the city of Murcia to be the most realistic as possible. From this map, we extracted a matrix of temperature, in °C, that corresponds to the Table 8. So, we defined a RoI of 5×5 cells covering the whole map indicated in Figure 11.

Given that the temperature data change gradually, and therefore new values are related to current ones, (for this specific examples it varies within the range of 28.6–30.2 °C), we have selected a representation matrix consisting in the variance divided by the average of this latter. Let us explain it by the Table 9.

Firstly we need to map the temperature measured in a previous moment before beginning all the gathering process. So, in Table 9, we have identified all the RoI cells by the pair (row, column) which corresponds to the first column, as well as, all these temperature values that correspond to the second column.

Taking these values, we calculated their variance, calculating also the average of them. Finally, we have built our representation matrix by dividing the variance per the calculated average. The result is the 3rd column of the table, but for the sake of seeing it as a matrix we present it in Table 10.

In order to prove that this representation matrix sparsifies the data, we have generated the last column of Table 9, which is the projection of the values of the last but one column with the values of the representation matrix.

**Figure 16:**
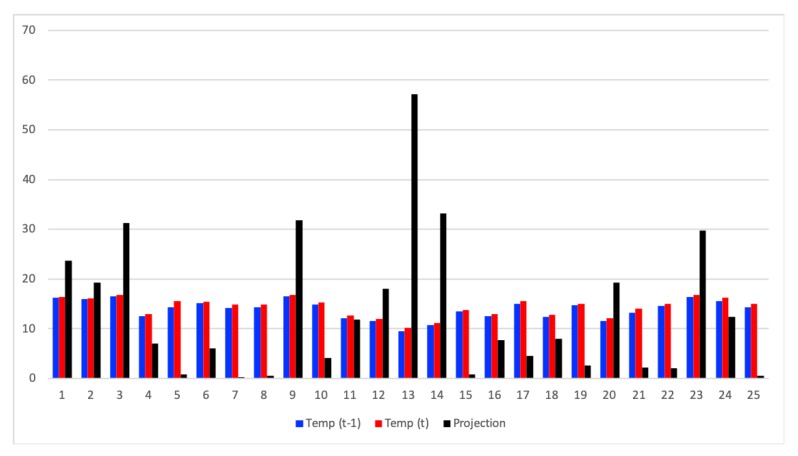
Temperature values obtained for previous and current time (t). Projection of current time temperature values on the sparsifying basis.

Figure 16 show the temperature values obtained in two different moments (t − 1 and t), as well as the projection of the values obtained in t onto the representation matrix. As we can see, only a few of this projection contain relevant information, fulfilling this way the sparsity requirement for the application of CS.

In this execution, the FC has received 51 data packets with some replicated information. So, in Table 11, we present the data in 18 rows without duplicates. Each data packet received by the FC corresponds to each row.

Let us focus on the first packet received by the FC, i.e., the first row of the table that we present in Table 12. The sequence of 0 s and 1 s corresponds to the cells of the RoI where nodes took the measurements. The first five elements correspond to the cells of the first row of the RoI, the second five elements to the second row, and so on (see Table 13). On the other hand, the rightmost column provides the projection values associated with the measures received by each node.

Although there are several cells with missing values like 4 (4, 1), 5 (5, 1), 6 (1, 2), 11 (1, 3), and 20 (5, 4) thanks to the use of CS we have obtained a reconstruction of the data as Table 14 presents.

So, observing both tables, we can conclude that the reconstructed data are close to the original values obtained by only the projections of the nodes.

**Figure 17:**
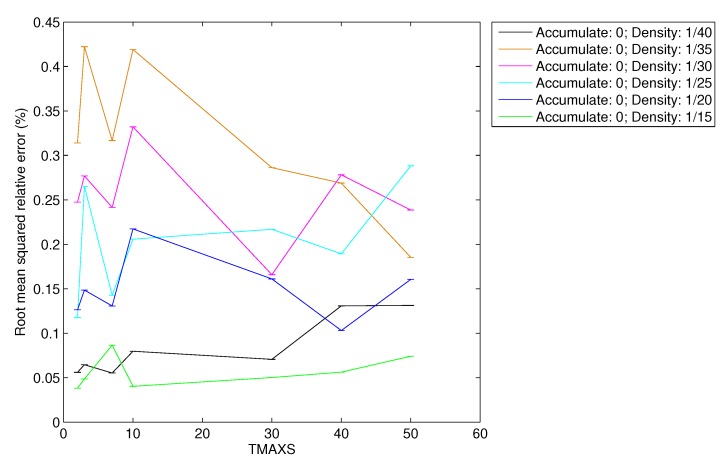
RMS relative error for CSPure.

**Figure 18:**
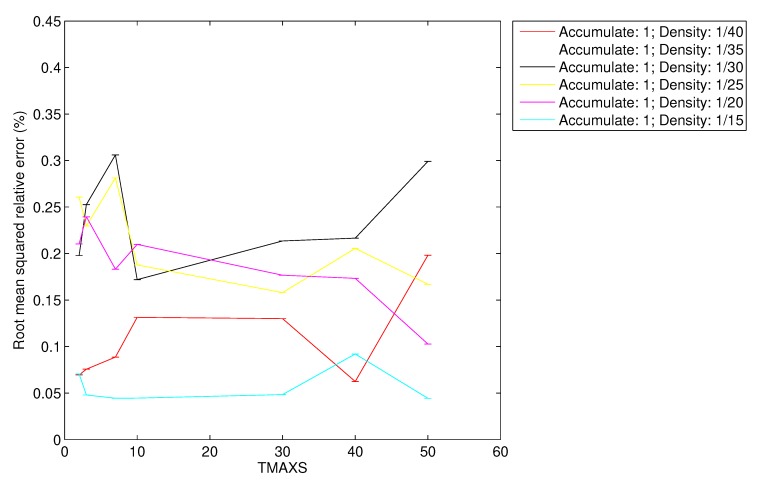
RMS relative error for CSAccum.

In Figure 17 and Figure 18 we present the root mean squared relative error (RelError) obtained for each simulated density of vehicles and varying also the TMAX parameter.

As we can observe, the RelError is lower than 0.45% for CSPure and 0.3% for CSAccum. In light of these results, we can also manifest the following two statements: the error in the reconstruction is independent of the TMAX parameter, and that RelError and the density of vehicles are related. This latter relation makes sense because the denser the scenario, the more data the FC receives, and therefore, the more accurate reconstruction it can obtain.

## 6. Conclusions and Future Work

In this paper, we have presented CS-VDH, a new harvesting protocol for VSNs oriented to obtain perishable information provided by vehicles thanks to their equipped sensors. This proposal comprises a geocast protocol to flood the query message issued by the FC into a specific RoI and whose format has been defined, the use of a VANET routing protocol for sending the measurements back to the FC, and finally the use of the CS technology, more specifically, the concept of projection to compress the information without hardly increasing the packet size at it traverses the network.

This solution outperforms previous proposals like DB-VDG in terms of overhead. Additionally, we have also compared our proposal with a variation without compression so as to check how efficient CS-VDH is in terms of the values that nodes can carry and the overhead they introduce. Although the NoComp variation can carry nearly up to one sensed value per packet more than our CS solution, the total overhead introduced by NoComp is higher than the CS variant. Moreover, the benefits of CS, where it is not necessary to receive all the values to accurately reconstruct them make the difference in terms of the amount of carried values negligible.

We have made the first analysis over our delay function and also studied the variations of its TMAX parameter, which sets the maximum time for nodes to answer to the FC with their sensed information. Due to the particularities of our scenario, we have seen that a TMAX value higher than 10 s ensures that CS-based schemes outperform the other alternatives in terms of delivered sensed values.

As future works, we are considering the application of CS in different urban scenarios. Another interesting task is that of changing the location of the Fusion Center, or even evaluate the use of more than one Fusion Center. The simulation of the deployment of gateways with access to the infrastructure network in the scenario can be another interesting future work. Finally, although we have used a representation matrix with the purpose of exemplifying the reconstruction process in CS, a study of different representation matrix is also desirable.

## Figures and Tables

**Table 1:** Summary of proposed compressed sensing (CS)-based solutions applied for data gathering in the WSN or Vehicular Sensor Network (VSN) domain. Legend: Theo. means that only a theoretical approach has been considered; Agg. approach means Aggregation or combination approach followed to include new information; Geocast means that a geocast method has been considered for the distribution of a message in a region of interest (RoI); Multi-hop unicast means that the proposed method considers a multi-hop mechanism to transmit the information towards a destination.

Proposals vs. Features	WSN	VSN	Theo.	Agg. Approach	Veh. Ferries	Geo Cast	Multi -Hop Unicast	Summary
Lee et al. [13] MOBEYES		X		No agg.			X	Proactive. Not practical due to packet size. No compression
Lee et al. [13] MOBEYES-ODH		X		No agg.		X	X	Reactive but network overload. No compression
Lee et al. [14] FleaNet		X			X		X	Only 1-hop query distribution. No compression
Lee et al. [15] VITP		X		Piggybacking			X	Packets increase size. No compression
Palazzi et al. [16]		X		Piggybacking		X	X	Two phases: geocast and gathering. No compression
Haupt et al. [5]	X			In the air				Only considered the signal plane
Chou et al. [23]	X			CS				Two phases: - 1st no-compression. - 2nd CS for WSN
Zhang et al. [24]	X			Adaptive CS				For image sensor networks. Focus on CS
Zhou et al. [25]	X			CS over graphs				VSN are dynamic. Not applicable
Pacharaney et al. [26]	X			Clusters and CS				Cluster of static nodes
Valsesia et al. [27]	X			CS				Focused only in the application of CS
Yu et al. [28]		X	X	CS at intersections				Theoretical. No simulations. VSN mobility not considered
Huang et al. [29]		X		CS	X			Vehicles collect data as they move
Lin et al. [30]		X	X	CS				Focused again on CS

**Table 2:** CS: the combination of projections is possible because they do not overlap.

	Areas				Projection Performed
	1	2	3	4	
**Node1**	X	X			P1 = A∗$X_{1}$ + B∗$X_{2}$
**Node2**			X	X	P2 = C∗$X_{3}$ + D∗$X_{4}$
**Combin.**	**X**	**X**	**X**	**X**	**P1+P2**

**Table 3:** CS: combination of data not possible because projected data overlap according to the boolean vector.

	Areas				Values
	1	2	3	4	
**Node1**	X	X	X		P1 = A∗$X_{2}$ + B∗$X_{2}$ + C∗$X_{3}$
**Node2**			X	X	P2 = C∗$X_{3}$ + E∗$X_{4}$
**Combin.**	**Not possible**				

**Table 4:** Parameters selection fo the different series in the graph.

	Param $p_{1}$	Param $p_{2}$	Param $p_{3}$
**Series 1**	20	2000	500
**Series 2**	20	2000	80
**Series 3**	20	1000	80

**Table 5:** Delay time(s) obtained for the three selected series and for 10 randomly selected distances to the fusion center (FC).

**Distance to FC (m)**	113	457	1649	1255	2318	2275	1113	1205	192	1417
**Series 1 (s)**	19.55	19.11	12.18	15.96	3.02	3,53	16.88	16.31	19.47	14.65
**Series 2 (s)**	20.00	20.00	19.75	20.00	0.00	0.00	20.00	20.00	20.00	19.99
**Series 3 (s)**	20.00	19.98	0.00	0.00	0.00	0.00	0.33	0.00	20.00	0.00

**Table 6:** Detailed information about the different traffic densities generated for the simulations.

Name	Density (veh/route/s)	veh/s	Total Injected Vehs
50	1/50	0.4	178
45	1/45	0.44	197
40	1/40	0.50	222
35	1/35	0.57	254
30	1/30	0.67	296
25	1/25	0.80	356
20	1/20	1.00	445
15	1/15	1.33	593
10	1/10	2	890

**Table 7:** Control overhead (total num. of generated messages) of both proposals.

Density	DBVDG-sbss	DBVDG-dbss	CS-VDH-NOCS	CS-VDH-CS
50	563,728.4	514,996.4	735.7	513.9
45	712,439.3	592,476.4	274.5	243.7
40	882,573.4	685,165.7	840.7	669.4
35	786,394.4	597,975.6	2390.6	2080.1
30	1,007,816.0	803,683.1	2261.3	2022.6
25	1,127,205.2	878,429.9	2216.6	1924.2
20	1,114,846.7	905,167.5	3239.5	2725.2

**Table 8:** Table representing a RoI of the scenario which is divided into 5 rows and 5 columns. Each of the cell corresponds to a specific area of the scenario whose real values is represented in °C.

Original Data				
30.00	29.80	29.90	30.20	30.00
29.70	29.60	29.20	29.00	29.70
28.90	28.60	28.90	29.00	28.90
29.50	29.80	29.90	30.00	29.50
29.80	29.90	30.10	30.00	29.80

**Table 9:** Table explaining the representation basis. Starting from the temperature taken in each area of the RoI in the previous instant represented by (row, column), we generate the variance (2nd column), then in the 3rd column we divide the variance per the average of them. Finally, in the 5th column, we represent the temperature obtained in the current timestamp (4th column) multiplying them by the corresponding values of the 3rd column.

Matrix Cell (r,c)	Temp. (t − 1)	Variance	Variance/Avg (Variances)	Temp (t)	Projection
(1,1)	16.21	5.13113104	1.442784308	16.45	23.73380187
(1,2)	16.01	4.26505104	1.199257759	16.06	19.26007961
(1,3)	16.52	6.63165504	1.864705413	16.76	31.25246273
(1,4)	12.55	1.94546704	0.547031307	12.97	7.094996046
(1,5)	14.37	0.18079504	0.050836403	15.51	0.788472614
(2,1)	15.13	1.40469904	0.394976803	15.48	6.114240905
(2,2)	14.23	0.08133904	0.022871115	14.89	0.340550909
(2,3)	14.32	0.14077504	0.039583479	14.89	0.589398005
(2,4)	16.54	6.73506304	1.893781934	16.83	31.87234996
(2,5)	14.93	0.97061904	0.272921098	15.27	4.167505174
(3,1)	12.12	3.32989504	0.936308247	12.71	11.90047782
(3,2)	11.63	5.35829904	1.506659976	11.95	18.00458672
(3,3)	9.47	20.02383504	5.630352208	10.16	57.20437843
(3,4)	10.69	10.59372304	2.978769641	11.15	33.21328149
(3,5)	13.49	0.20684304	0.058160645	13.77	0.800872086
(4,1)	12.49	2.11644304	0.595106767	13.00	7.736387977
(4,2)	14.97	1.05103504	0.295532671	15.52	4.586667058
(4,3)	12.46	2.20463104	0.619903691	12.87	7.9781605
(4,4)	14.74	0.63234304	0.177803804	15.08	2.681281357
(4,5)	11.56	5.68727104	1.599161149	12.07	19.30187507
(5,1)	13.19	0.56972304	0.160196155	14.03	2.247552051
(5,2)	14.64	0.48330304	0.135896362	15.05	2.04524025
(5,3)	16.46	6.32623104	1.778825526	16.76	29.81311582
(5,4)	15.59	2.70668304	0.761071933	16.30	12.4054725
(5,5)	14.31	0.13337104	0.037501604	15.58	0.561023994

**Table 10:** Representation matrix to sparsify the measurements.

Representation Matrix				
1.4428	1.1992	1.8647	0.5470	0.0508
0.3950	0.0229	0.0396	1.8937	0.2730
0.9363	1.5067	5.6303	2.9788	0.0582
0.5952	0.2955	0.6199	0.1778	1.5991
0.1602	0.1359	1.7788	0.7610	0.0375

**Table 11:** Table of received data. The first column contains the values of the boolean vectors, and the second the projection performed.

Obtained Boolean Vector and Associated Projections																									
0	0	0	0	0	0	0	0	0	0	0	0	0	0	0	0	0	1	0	0	0	0	1	1	0	**50.1967**
0	0	0	0	0	0	0	0	0	0	0	0	0	0	0	0	0	1	1	0	0	0	0	1	0	**23.0649**
0	0	0	0	0	0	0	0	0	0	0	0	0	0	0	0	0	1	1	0	0	0	0	1	1	**23.6259**
0	0	0	0	0	0	0	0	0	0	0	0	0	0	0	0	0	1	1	0	0	0	1	1	0	**52.8780**
0	0	0	0	0	0	0	0	0	0	0	0	0	0	0	0	1	1	0	0	0	0	0	0	0	**12.5648**
0	0	0	0	0	0	0	0	0	0	0	0	0	1	0	0	0	1	1	0	0	0	0	1	1	**56.8392**
0	0	0	0	0	0	0	0	0	0	0	0	1	0	0	0	0	1	1	0	0	0	0	1	1	**80.8303**
0	0	0	0	0	0	0	0	0	0	0	1	0	0	0	0	1	1	0	0	1	1	0	0	0	**34.8622**
0	0	0	0	0	0	0	0	0	0	0	1	0	0	0	1	1	1	0	0	0	0	0	0	0	**38.3058**
0	0	0	0	0	0	0	0	0	0	0	1	0	0	0	1	1	1	0	0	1	0	0	0	0	**40.5533**
0	0	0	0	0	0	0	0	0	0	0	1	0	0	0	1	1	1	0	0	1	1	0	0	0	**42.5986**
0	0	0	0	0	0	0	1	0	0	0	0	0	0	0	0	0	0	0	0	0	0	0	0	0	**0.5893**
0	0	0	0	0	0	0	1	1	1	0	0	0	1	1	0	0	0	0	0	0	0	0	0	0	**127.0469**
0	0	0	0	0	0	1	1	0	0	0	1	0	0	0	1	1	0	0	0	0	0	0	0	0	**26.8925**
0	0	0	0	0	0	1	1	0	0	0	1	0	0	0	1	1	0	0	0	1	0	0	0	0	**46.1944**
0	1	1	0	0	0	1	1	0	0	0	0	0	0	0	0	0	0	0	0	0	0	0	0	0	**51.4425**
1	0	0	0	0	1	1	1	0	0	0	0	0	0	0	0	0	0	0	0	0	0	0	0	0	**30.7780**
1	0	0	0	0	1	1	1	1	0	0	0	0	0	0	0	0	0	0	0	0	0	0	0	0	**62.6503**

**Table 12:** Boolean vector obtained from the first row of Table 11 which contains 25 values corresponding to the 25 areas of the defined RoI.

Boolean Vector Received by One Node																								
0	0	0	0	0	0	0	0	0	0	0	0	0	0	0	0	0	1	0	0	0	0	1	1	0

**Table 13:** Boolean vector from Table 12 transformed to a matrix to be easily mapped to the cells of the RoI.

0	0	0	0	0
0	0	0	0	0
0	0	0	0	0
0	0	1	0	0
0	0	1	1	0

**Table 14:** Table of reconstructed data.

Reconstructed Data				
30.10	29.95	29.87	30.31	30.09
29.65	29.55	29.18	29.02	29.44
29.31	28.83	28.98	29.13	29.02
29.36	29.79	29.86	29.95	29.97
29.77	29.83	30.10	30.05	29.78

## Abbreviations

The following abbreviations are used in this manuscript:

CS	Compressed Sensing
CS-VDH	Compressed-Sensing-based Vehicular Data Harvesting
DB-VDG	Delay-Bounded Vehicular Data Gathering
DTN	Delay Tolerant Network
FC	Fusion Center
ns2	network simulator 2
RoI	Region of Interest
VITP	Vehicular Information Transport Protocol
VANET	Vehicular Ad-hoc Network
VSN	Vehicular Sensor Network
WSN	Wireless Sensor Network
